# The effect of written material and verbal method education on anxiety and depression in patients with myocardial infarction in selected hospitals in Iran

**Published:** 2014-10

**Authors:** NADER AGHAKHANI, KAMAL KHADEMVATAN, MOHAMMAD REZA DEHGHANI

**Affiliations:** 1 Urmia University of Medical Sciences, Urmia, Iran; 2 Quality improvement in clinical education Research Center, Shiraz University of Medical Science, Shiraz, Iran;

**Keywords:** Verbal, Education, Anxiety, Depression, Myocardial infarction

## Abstract

**Introduction:** Myocardial infarction (MI) is the damage to the heart muscle, or myocardium, resulting from the lack of blood flow to the heart. MI patients experience mental and emotional problems such as depression and anxiety. These complications could cause delay in resuming work, decreased quality of life and increased risk of death. The role of education in facilitating adaptation is very important in these patients. The purpose of this study was to determine the effect of written material and verbal method education on anxiety and depression in patients with myocardial infarction in Urmia hospital in 2009.

**Methods:** This study was a quasi-experimental study, comparing the effect of education on anxiety and depression in patients with myocardial infarction in hospitals affiliated to Urmia University of Medical Science. 124 patients were selected randomly and divided into two groups. The experimental group was educated through face to face training and an educational booklet (Written Material and Verbal Method). The control group did not receive any intervention. The level of anxiety and depression was evaluated, using HADS questionnaire at 3 intervals: after 48 hours of admission, the discharge day and 2 months after discharge.

**Results:** The findings suggested that MI patients were worried about their social role, interpersonal relations and personal health. Such problems can aggravate symptoms and complicate the future care. There was no significant difference between the control and experimental groups before the intervention, but after the intervention, anxiety and depression in the experimental group was significantly less than that in the control group (p<0.05).

**Conclusion:** Considering the beneficial results obtained from written materials and verbal method education on reducing anxiety and depression in cases with myocardial infarction, this may be one of the health care goals. More research on more patients is required to achieve more conclusive results.

## Introduction

One of the major causes of death worldwide is myocardial infarction that is a chronic disease and may be a major catastrophic event leading to sudden death or hemodynamic deterioration (1).

Some complications such as depression and anxiety in patients recovering from a myocardial infarction are common and may act as barriers in their lifestyle decreasing the patients’ health-related quality of life. Depression and depressive symptoms are common in patients with acute myocardial infarction (AMI) although the prevalence varies depending on the assessment method (2, 3).

Based on WHO estimation, in developing countries, coronary artery diseases including myocardial infarction are a major public health problem (4).

Myocardial ischemia and the possibility of fatal dysrhythmias increase the level of stress, affect patients' learning and ability to maintain information and decrease life quality, thus endangering the adjustment process and future morbidity of discharged patients (5).

At different time periods after hospitalization, patients may experience different worries and fears (6).

Several recent studies have reported that the symptoms of depression and anxiety which arise in patients with MI may predict the subsequent mortality in them (7).

It is reported by the pooled results of a recent study that 15–20% of patients have major depression, and a similar proportion have minor depression (6).

Recent studies on depression and anxiety confirm that the existence of psychological problems before and after MI increase the risk of cardiac mortality (8).

Health professionals agree that comprehensive information in a format understandable to the patient is of utmost importance. These formats can be verbal and written information. The patients may be disempowered if they are unable to use the information or forget what they have been told after discharge (9).

The aim of this study was to determine the effect of written materials and verbal method education on anxiety and depression in patients with myocardial infarction in selected Iranian hospitals in 2009. 

## Methods

This study was a quasi-experimental study comparing the effects of written materials and verbal method education on anxiety and depression in patients with myocardial infarction in the selected hospitals affiliated with Urmia University of Medical Sciences. 124 eligible patients were selected randomly as the subjects of the study. They were then divided into a control and a test group. The experimental group underwent face to face training and was provided with an educational booklet. Some systematic reviews suggested that written materials in the form of summary letters written to the patient by the physician or information booklets were effective patient education strategies with respect to satisfaction and information recall. The written material should, of course, be prepared at a reading level suitable for the general population. Written information in the form of a new patient information booklet improved patient knowledge and reduced his/her confusion (9).

Levels of anxiety and depression were evaluated in patients at three intervals: 48 hours after admission, the day of discharge and two months after discharge, using the HADS questionnaire.

The HADS questionnaire consists of 14 items: seven items on depression (HADS-D) and seven items on anxiety (HADS-A). HADS is formulated in a language that is readily understandable. To increase the acceptability, symptoms of severe psychopathology were omitted. Likewise, symptoms that can be caused by somatic illnesses were not included. HADS is well accepted in somatically ill patients, population settings and oncology cases. HADS-D focuses mainly on the reduced pleasure response aspect of depression, whereas HADS-A focuses mainly on generalized anxiety issues of worry and fears of the future, with one item for panic attacks. Each item is scored from 0 (minimally present) to 3 (maximally present), and the sum score on each subscale (HADS-A and HADS-D) ranges from 0 to 21. Among the several instruments designed for psychiatric diagnoses, the Hospital Anxiety and Depression Scale (HADS) distinguishes itself from all other scales due to its ability to assess anxiety and depression without investigating somatic symptoms. HADS is often used to analyze a variety of diseases in the clinical setting.  This method was developed in 1983 and consists of a series of 14 questions, 7 of which are related to anxiety (HAD-A) while the other 7 questions are related to depression (HAD-D). The creators  of the scale considered a score of less than 8 suggesting lack of any mental disorder, a score equal to or greater than 8 suggesting a disorder be “probably” present, while a score above 10 was considered to indicate that a patient was “highly likely” to have a disorder.

HADS has been used to help diagnose patients suffering from chest pain, atypical chest pain and heart disease.  

We excluded patients with clinical conditions instability. In addition, patients were excluded if diagnosed with any psychiatric disorders that cause changes in their awareness.

If a patient presented a limitation in completing the HADS, such as difficulty reading, the health professional would offer assistance. The health professionals encouraged the patients to choose answers based on symptoms they had experienced during the previous week and asked them to provide spontaneous answers without excessive reflection.

At the beginning of the study, no significant difference was found between the intervention and control groups, using a repeated measures analysis of variance. The current study was approved by the Ethics Committee consistent with the terms of the Helsinki Declaration. A bidirectional model was used for statistical analysis with a 5% level of statistical significance. The SPSS 13, was used for all analyses. A reliability analysis of the HADS total (all items) and HADS scores was conducted to ensure that the measures satisfied the criteria for clinical and research purposes, using the Cronbach‘s alpha statistical procedure. A Cronbach's alpha reliability statistic of 0.70 was considered as the minimum acceptable criterion of instrument internal reliability. 

## Results

The sample (n=124) consisted primarily of middle-aged patients (Mean±SD=55±2.6), females outnumbering males (53.3%). The majority of the patients were married (93.3%) and lived with their families (73%). Annual income was less than 3000 dollars for 80% of the participants, and 40% had less than high school education. For statistical comparisons, the educational level and annual income variables were dichotomized to below high school level and high school level or above for education and less than and more than 3000 US dollars for annual income. 78.5% of patients were sick between 0-3 years .

In a study of patients who had suffered an AMI, the occurrence of high levels of depressive symptoms was measured by the Beck Depression Inventory. In numerous studies, depression has been associated with mortality, and physical disability. In addition, depressive symptoms are associated with use of healthcare resources and lack of lifestyle changes (6).

High levels of anxiety symptoms, as measured by the Hospital Anxiety and Depression Scale (HADS), in a general population, have been reported in the beginning of hospitalization.

Other studies, using the same instruments, have reported the presence of anxiety symptoms in patients with AMI 3 months after discharge, but the level was less in educated individuals (p<0.05).

Stress was significantly and positively correlated with age and gender and marital status (0.003), but negatively correlated with income, and re-hospitalization for cardiac events. There was no significant difference between the control and experimental groups before the intervention, but after the intervention, anxiety and depression in the experimental group was significantly less than that in the control group at the discharge time and 3 months after discharge from hospital (p<0.05). 

## Discussion

The present study examined the relationship between depression and anxiety in the hospital and mortality 12 months after MI. In addition, the study was concerned with   the level of anxiety and depression, using HADS questionnaire at 3 intervals: 48 hours after admission, on the discharge day and 2 months after discharge. After the MI, patients usually suffer psychological complications, and physical symptoms, with adverse effects on everyday life, and more use of health resources.

The findings of this review are important because of the fact that providing information to patients on discharge from hospital to home is an essential component of quality care provision for the majority of clinical staff and is a fundamental right of all patients being discharged.

The results of this review found that providing written and verbal health information, when compared to verbal information only, does significantly increase the knowledge of parents of children who have been discharged from hospital to home. It is important to note that the knowledge scores in both trials may have been underestimated. This is suspected because there was no requirement in either trial that the person who was given the discharge information participated in the follow-up interviews. This could have resulted in a parent participating in the follow-up interview(s), and not being the parent who was provided with the original written and verbal discharge information.

One study, conducted among MI patients in Canada, revealed that both major depression and elevated BDI scores predicted cardiac mortality 6 months after acute MI regardless of the severity of infarction. By the 18-month follow-up, 21 patients had died, 19 (8.6%) due to cardiac causes. Again, both major depression and elevated BDI scores were related to cardiac mortality although only the BDI prediction remained statistically significant when other clinical variables, such as previous MI were controlled. In the same study, anxiety also emerged as a predictor of recurrent cardiac events independent of depression (10).

In general, depression and anxiety may substantially reduce a person’s quality of life and that of his or her family.

Symptoms of depression and anxiety measured in the hospital 2 to 15 days after MI did not predict 12-month mortality. This finding is clearly at odds with some, although not all, previous research. Because studies have varied in location, patient population, sample size, and the means of measuring depression, some variation in results is hardly surprising. However, in another analysis that included only patients who died after discharge, symptoms of depression and anxiety failed to predict mortality (11).

Some studies have reported a significant association between depression, anxiety, and complications after MI. It could seem to predict mortality in studies in which disease severity is significantly correlated with depression and anxiety (12).

As in the present study, depression and anxiety were not related to mortality, either at 6 or 18 months. Thus, it remains possible that depression and anxiety are markers of disease severity that is the underlying cause of death (13).

Anxiety and depression would appear to predict clinical prognosis of MI mainly in studies that have not controlled whether disease severity is significantly correlated with depression or anxiety (14).

Others have noted that one of the main obstacles to attributing a causal role to mood status in clinical prognosis after MI is the potential confounding of mood after MI with disease severity. In many of the studies, a significant association was found between mood and mortality, but the association was no longer statistically significant after intervention for disease severity (15).

We used a verbal and written format for educating the patients. This method decreased the depression and anxiety in them. A systematic review of two trials comparing written and verbal information with verbal information only found that knowledge significantly improved when written materials were combined with verbal health information (16).

This study found that providing written information plus verbal information was more effective in improving knowledge for the patients discharged from hospital. Of course, generalizability of the results of this systematic review is guarded. We cannot state that the findings are appropriate to the patients providing their own care. 

## Conclusion

The study findings showed the difficulties that patients experience in the period after MI. Immediately after discharge, health care systems should consider patients' needs for psychological support and educational needs. To provide effective psychological interventions, counseling should include comprehensive assessment of anticipated difficulties and worries that postpone adjustment. Identification of individual concerns is essential so that counseling and teaching can be suitable to the patients’ needs.

More attention should be paid to the cultural aspects of patient teaching in discharge planning. Adequate teaching is needed to decrease patient uncertainty about issues needed for successful relaxation. The importance of written instructions and recommendations should be emphasized to change stressful behaviors. The significance of exercise participation and being relaxed for improving patients' health should be stressed. Educative-counseling programs should be based on the needs of MI patients. 

Reduction of depression and anxiety by written materials and verbal method education has many benefits in recovery from disease and control of other adverse outcomes. Replication of the study by using a larger sample and longitudinal design and other educational methods are recommended. 
